# Metabolic self-feeding in HBV-associated hepatocarcinoma centered on feedback between circulation lipids and the cellular MAPK/mTOR axis

**DOI:** 10.1186/s12964-024-01619-5

**Published:** 2024-05-21

**Authors:** Ying Zhu, Yingke Zhao, Zhouyu Ning, Yong Deng, Bing Li, Yun Sun, Zhiqiang Meng

**Affiliations:** 1 Minimally invasive therapy center, Shanghai Cancer Center, Fudan University Shanghai Cancer Center, Shanghai, 200032 China; 2 Department of Research and Development, Department of Nuclear Medicine, Shanghai Proton and Heavy Ion Center, Fudan University Shanghai Cancer Center, Shanghai, 201321 China; 3 Cancer Institute, Fudan University Shanghai Cancer Center, Shanghai, 200032 China; 4 Shanghai Key Laboratory of radiation oncology (20dz2261000), Shanghai, 201321 China; 5 Shanghai Engineering Research Center of Proton and Heavy Ion Radiation Therapy, Shanghai, 201321 China; 6 Eye Institute, Eye and ENT Hospital, College of Medicine, Fudan University, Shanghai, China

**Keywords:** Hepatitis B Virus (HBV), Hepatocellular carcinoma (HCC), Multi-omics, Lipid metabolism, Metabolic self-feeding, MAPK/mTOR

## Abstract

**Introduction:**

Hepatitis B Virus (HBV) is widely recognized as a “metabolic virus” that disrupts hepatic metabolic homeostasis, rendering it one of the foremost risk factors for hepatocellular carcinoma (HCC). Except for antiviral therapy, the fundamental principles underlying HBV^−^ and HBV^+^ HCC have remained unchanged, limiting HCC treatment options.

**Objectives:**

In this study, we aim to identify the distinctive metabolic profile of HBV-associated HCC, with the promise of identifying novel metabolic targets that confer survival advantages and ultimately impede cancer progression.

**Methods:**

We employed a comprehensive methodology to evaluate metabolic alterations systematically. Initially, we analyzed transcriptomic and proteomic data obtained from a public database, subsequently validating these findings within our test cohort at both the proteomic and transcriptomic levels. Additionally, we conducted a comprehensive analysis of tissue metabolomics profiles, lipidomics, and the activity of the MAPK and AKT signaling pathway to corroborate the abovementioned changes.

**Results:**

Our multi-omics approach revealed distinct metabolic dysfunctions associated with HBV-associated HCC. Specifically, we observed upregulated steroid hormone biosynthesis, primary bile acid metabolism, and sphingolipid metabolism in HBV-associated HCC patients’ serum. Notably, metabolites involved in primary bile acid and sphingolipids can activate the MAPK/mTOR pathway. Tissue metabolomics and lipidomics analyses further validated the serum metabolic alterations, particularly alterations in lipid composition and accumulation of unsaturated fatty acids.

**Conclusion:**

Our findings emphasize the pivotal role of HBV in HCC metabolism, elucidating the activation of a unique MAPK/mTOR signaling axis by primary bile acids and sphingolipids. Moreover, the hyperactive MAPK/mTOR signaling axis transduction leads to significant reprogramming in lipid metabolism within HCC cells, further triggering the activation of the MAPK/mTOR pathway in turn, thereby establishing a self-feeding circle driven by primary bile acids and sphingolipids.

**Supplementary Information:**

The online version contains supplementary material available at 10.1186/s12964-024-01619-5.

**Scheme 1:**
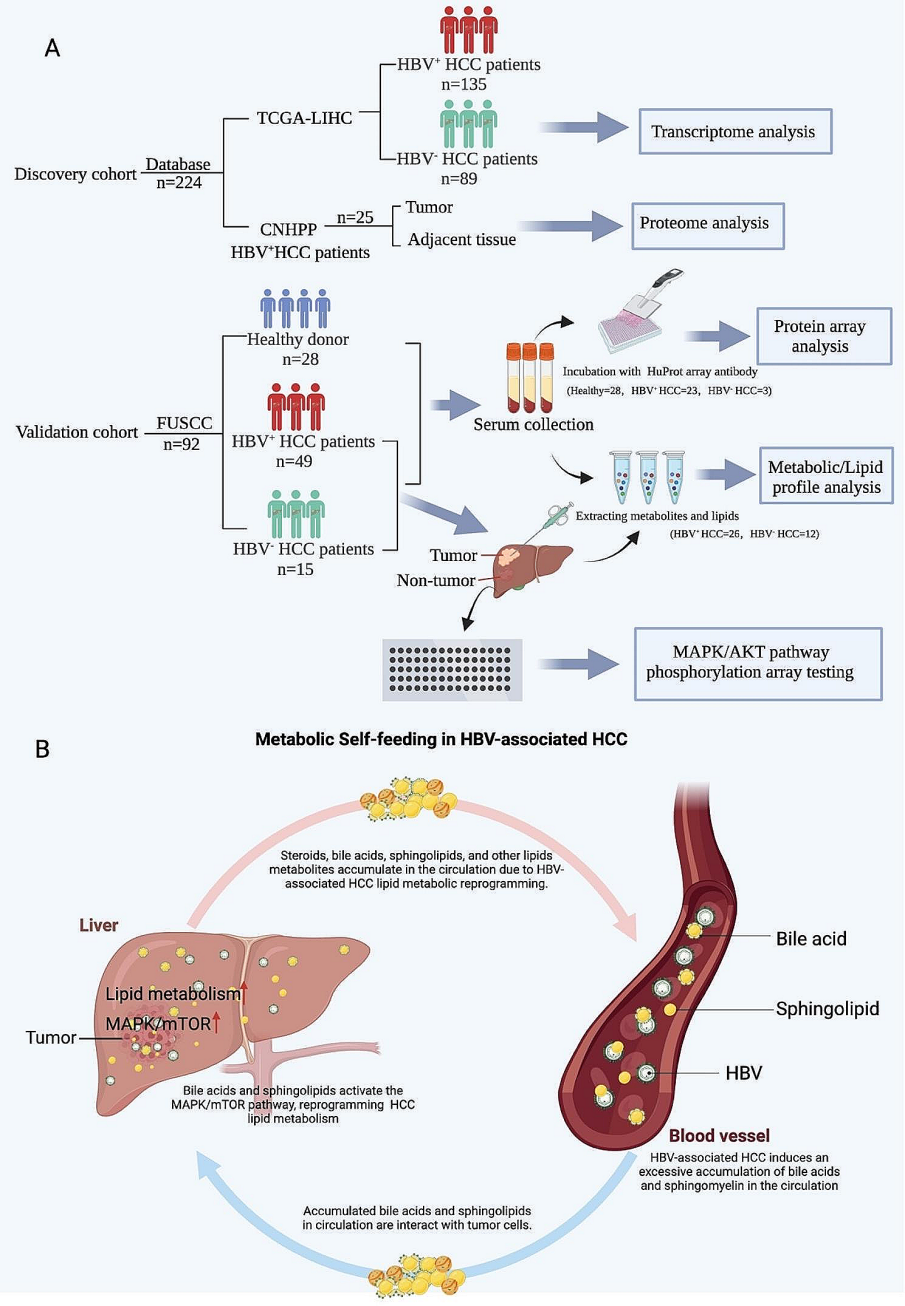
Schematic diagram of the study. (**A**) The study’s design first utilized transcriptomics and proteomics data from a public database to compare the metabolic profiles of HBV^+^ and HBV^−^ HCC patients. Our cohort’s proteome and metabolome were examined to validate the dysregulated metabolic pathways. Next, the samples were examined with an antibody array to confirm the underlying mechanism. (HCC: hepatocellular carcinoma, FUSCC: Fudan University Shanghai Cancer Center) (**B**) The metabolic self-feeding in HBV-associated HCC

## Introduction

Despite substantial progress in the clinical management of hepatocellular carcinoma (HCC), its therapeutic efficacy remains relatively limited, making it one of the most prevalent malignancies and a leading cause of tumor-related mortality globally [1, 2]. Hepatitis B Virus (HBV), a representative DNA virus, is widely acknowledged as a significant risk factor for HCC development, particularly in Asia, where it contributes to approximately 85% of HCC cases [3]. HBV infection exerts extensive effects on host cell metabolism, earning the designation of a “metabolic virus” due to its disruptive impact on cellular metabolic processes [4, 5].

Recently, it has become evident that HBV infection disrupts cellular metabolic processes and facilitates the progression of HCC through multiple mechanisms [6]. The integration of viral DNA into the host genome dysregulates critical signaling pathways involved in cell growth and proliferation [7]. As a result, the physiopathological characteristics of HBV-associated HCC differ from those of HBV- non-associated HCC. However, most studies on the distinctions between HBV-associated HCC and non-associated HCC have primarily focused on clinical and etiobiological features. For instance, one study demonstrated that HBV-associated HCC has a poorer prognosis than non-associated HCC [8]. Additionally, significant differences have been found between HBV-negative and positive HCC in terms of gene expression [9], DNA methylation [10], and protein modifications [11]. While some studies have explored the metabolic differences through combined genomics and proteomics analysis in tumor tissues and non-tumor tissues from HBV-positive patients, investigating metabolomics and lipidomics differences between HBV-positive and negative patients remains relatively limited [12]. Given the substantial impact of HBV on HCC metabolism, there is an urgent and imperative requirement to integrate genomics, proteomics, metabolomics, and lipidomics in a comprehensive exploration of the metabolic variances between HBV-positive and -negative HCC for individualized treatment strategies and offering potential targets for therapeutic interventions.

Therefore, in this study, we first conducted a comprehensive analysis of HCC using publicly available databases, such as the Cancer Genome Atlas Project (TCGA) and the Chinese Human Proteome Project (CNHPP) Liver Data Portal, to explore the transcriptional and proteomic profiles of 224 patients. To further validate our findings, serum protein profiles of 92 hepatocellular carcinoma patients from our hospital were analyzed using protein microarrays, and serum metabolite profiles were examined using ultra-performance liquid chromatography-electrospray ionization/quadrupole time-of-flight mass spectrometry (UPLC-ESI-Q-TOF-MS) to summarize the metabolomic profiles. Moreover, biopsy tissues collected from HBV-positive and HBV-negative HCC patients were analyzed to validate metabolic and lipidological profiles, and relevant metabolic signaling cascades were examined in situ. By adopting this multi-omics approach, our research endeavors to accurately discern the metabolic profiles of HBV-positive and HBV-negative HCC and offer a more personalized and efficacious therapeutic strategy for clinical applications.

## Materials and methods

### Patient recruitment and sample collection

Ninety-two participants who sought medical care at the Department of Integrative Medicine in Fudan Affiliated Cancer Center were enrolled in this study between January 1, 2016, and May 31, 2017. Among them were 28 healthy controls, 49 patients with HBV^+^ hepatocellular carcinoma (HCC), and 15 with HBV^−^ HCC. The characteristics of the patients are summarized in Table S1. To mitigate the potential impact of gender on metabolites, only male participants were included in this study. Patients’ information, serum, plasma, and biospecimens were collected from the Minimally Invasive Therapy Center at Fudan University Shanghai Cancer Center, following informed consent and approval from the Institutional Review Board (IRB) (No. 1904200-13). Exclusion Criteria: (1) Individuals who have consumed any medications within 48 h prior to sample collection that could potentially alter proteomic or metabolomic analysis outcomes will be excluded. (2) Subjects presenting with significant comorbidities or health concerns, including but not limited to active infections or severe cardiovascular conditions, will be excluded from this study. (3) Participants diagnosed with liver diseases of a different etiology, such as non-alcoholic fatty liver disease or autoimmune liver diseases, will not be included. (4) Individuals with concurrent malignancies of any type will be excluded. (5) Patients undergoing treatments known to affect liver function or influence the progression of hepatocellular carcinoma, including specific antiviral or chemotherapy agents, will be excluded from participation. (5) Patients with HCV positive will be excluded from participation.

Blood samples were obtained using anti-coagulation tubes containing potassium-EDTA (Becton et al.) and promptly transported to the laboratory for further processing. Serum was prepared by collecting blood in a serum separator tube, followed by centrifugation at 1000 g for 10 min at 4 degrees Celsius after clotting. Plasma was obtained by collecting blood in purple top EDTA tubes and subjecting them to centrifugation at 2000 rpm for 20 min at 4 degrees Celsius. The resulting supernatants were carefully collected, divided into aliquots, and stored at -80 degrees Celsius for subsequent analysis. Biopsies were acquired using an 18G diagnostic needle and preserved in MACS® Tissue Storage Solution (Miltenyi Biotec) at -80 degrees Celsius for future use.

### Data source and bioinformatics analysis

Data for this study were obtained from two sources: the TCGA-Liver Hepatocellular Carcinoma (HCC) cohort (https://portal.gdc.cancer.gov/projects/TCGA-LIHC) and the CNHPP (Chinese human proteome program) liver data portal (http://liver.cnhpp.ncpsb.org/). The gene expression data, consisting of raw count values, was collected from the TCGA dataset, which included 135 cases of HBV^+^ HCC and 89 cases with no history of HBV infection.

Transcriptional profiling was performed using the R package “edgeR.” The gene expression data at the mRNA, proteome, and phosphoproteome levels were subjected to t-tests. The TB tool was utilized to visualize genes with a false discovery rate (FDR) of less than 0.1 and a fold change greater than 1.5. Pathway enrichment analysis and biological function annotation were conducted using the Kyoto Encyclopedia of Genes and Genomes (KEGG).

Differential gene expression analysis was performed using the “limma” package, and Gene Set Enrichment Analysis (GSEA) was carried out using the cluster file R package, with a significance threshold of *p* < 0.05. Data for significant metabolism pathway gene sets were obtained from the Molecular Signatures Database (MSigDB, https://www.gsea-msigdb.org/) and KEGG. The activity levels of HCC-related pathways were calculated using Gene Set Analysis (GSA). Additionally, a Kaplan-Meier survival curve was constructed based on the log-rank test to assess differences in prognosis of corresponding metabolic pathways or metabolites.

### Protein microarray fabrication and data processing

In our serum proteomics analysis, we included samples from 28 healthy individuals and 26 patients diagnosed with hepatocellular carcinoma (HCC), which comprised 23 patients with HBV-positive HCC and 3 with HBV-negative HCC. The HuProtTM array was constructed from a protein library of over 20,000 unique human open reading frames using the Saccharomyces cerevisiae expression system; each recombinant protein had a GST-His6 tag at the N-terminal [13]. The serum, after dilution, was added to the array surface and subsequently washed with Tris-buffered saline containing 0.1% Tween 20 detergent (TBS-T) 3 times after incubation for 1 h. Proceed to the secondary antibodies (anti-human IgG, IgM), after incubation the array was washed and dried in a sliding washer (Capital et al., Beijing, China), and the array was washed and detected with a microarray scanner (Capital et al., Beijing, China). The raw data, defined as the median foreground and background intensities of every spot in the microarray, were downloaded from Scanner. The ratio of foreground to background was regarded as the raw intensity of the spot. The mean signal-to-noise ratio (SNR) was used to represent the signal of the protein. The normalization was done by normalizing between arrays package in limma [14]. Differentially expressed markers with fold change ≥ 1.2 and *p* < 0.05 were identified as protein candidates and recruited for further investigation.

### Metabolomic analysis

#### Serum metabolites separation

Furthermore, to corroborate our proteomics findings, metabolomics assessments were conducted on a group of 36 HCC patients, including 26 with HBV-positive HCC and 10 with HBV-negative HCC. Serum samples (200 µL) were extracted with 800 µL of 1:1 (v/v) acetonitrile/methanol. The samples were vortexed and sonicated for 5 min in an ice-water bath. After protein precipitation and centrifuge, the supernatant was collected and dried by vacuum freeze drier (FD-1 A-50, Bilon, Shanghai). Reconstituted with 200 µL acetonitrile/water (1:1, v/v), then processed to LC-MS analysis. Detection of metabolite profile was performed by an AB SCIEX Q-TOF mass spectrometer (X500B) coupled to an ExionLCTM-Ultra High-Performance Liquid Chromatography (UHPLC) system (AB et al.) in both positive and negative ionization. To evaluate the repeatability and robustness of the instrumental system, the quality control process identical to that of the metabolomic analysis was also applied, injected every 6 samples. All samples above were maintained at 4 °C during preparation and analysis.

### Tissue metabolomics and lipidomics sample extraction

Tissue metabolite extraction was performed according to the methods described in a previous publication [15]. Approximately 10 mg of biopsy tissue was homogenized with 1.5 mL of prechilled methanol/water for 5 min. The resulting mixture was centrifuged at 10,000 g for 10 min at 4 °C, and the supernatant was collected as the aqueous extract. The remaining pellet was used for the extraction of organic compounds. The pellet was treated with 1.6 mL of prechilled dichloromethane/methanol (3:1) to extract the organic metabolites. After homogenization, the mixture was centrifuged at 10,000 g for 10 min, and the supernatant was collected as the organic extract.

For lipidomic sample preparation, the biopsies were subjected to liquid-liquid extraction using MTBE/MeOH/H2O [16]. Each sample was treated with 300 µL of cold MeOH, followed by homogenization for 2 min. Subsequently, 1 mL of MTBE was added to the mixture, which vibrated and equilibrated at 4 °C for 10 min. The resulting supernatant, obtained after centrifugation, was collected for further analysis.

### UPLC-Q-TOF-MS instrumentation and measurement conditions

Hydrophilic interaction liquid chromatography (HILIC) and reverse-phase liquid chromatography were adopted. A Waters XSelect HSS T3 C18 column (2.1*150 mm, 2.5 μm) was adopted for reversed-phase separation, while the autosampler and column oven temperature was maintained at 4 °C and 40 °C, respectively. Mobile phases for C18 consisted of 2 mM ammonium acetate in 0.05/99.95 formic acid/water (A) and 1/1 acetonitrile/isopropanol (B). The column was eluted at 0.3 ml/min using a 5-100% phase B, and the injection volume was 1µL. An ACQUITY UPLC BEH hylic column (2.1*100 mm, 1.7 μm, Waters, Milford, MA, USA) was adopted. The column elution process was run at 0.3 mL/min with 10 mM ammonium acetate in 0.1/99.9 formic acid/water as phase A and 95/5 acetonitrile/water containing 10 mM ammonium acetate and 0.1% formic acid as phase B. The autosampler and column oven temperatures were maintained at 4 °C and 45 °C, and the injection volume for all samples was 2 µL.

A reversed-phase C18 column (2.1 mm*100 mm, 2.6 μm, Kinetex) was used for the chromatographic separation of lipids. The column temperature was kept at 55 °C. A gradient of (A) water/acetonitrile (40:60, v/v) and (B) acetonitrile/2-propanol (10:90, v/v), both containing 10 mM ammonium formate at a flow rate of 0.3 mL/min was used. The elution gradient started with 32% B, then linearly increased to 85% B at 15.5 min, then 97% B at 15.6 min, and held for 2.4 min. The gradient was back to 32% B at 18.1 min. The temperature of the sample manager was kept at 10 °C. The mass spectrometer was operated with a capillary voltage of 3.5 kV in positive mode and 3.0 kV in negative mode. The capillary temperature was set as 300 °C. Aux gas heater temperature was 350 °C. IDA auto-MS mode was used for MS/MS spectra collection. Using strategies, such as comparing the separated peaks from previous MS runs, mass matches from information provided by publicly available databases, and molecular structure prediction based on relationships between metabolites and their corresponding M2 features. The metabolite identification was accomplished. The lipids were identified by automated comparison to the chemical reference library standard via the software MS-DIAL 4.38.

### Antibody array

Protein extraction from the tissue was performed using RIPA buffer supplemented with proteinase inhibitors. The tissue was homogenized, and the resulting mixture was centrifuged at 13,000 g for 20 min at 4 °C. The supernatant containing the soluble protein fraction was collected. To assess the protein expression of the MAPK/AKT/mTOR signaling pathway, a Human phosphorylation array C1 (RayBio/AAH-AKT-1-8) was utilized. The raw data, representing the median foreground and background intensities of each spot on the microarray, were obtained from the scanner. The foreground-to-background intensity ratio was considered the raw intensity of each spot.

## Results

### Multi-omics validation of HBV-induced alterations in lipid metabolism in HCC

**Fig. 1:**
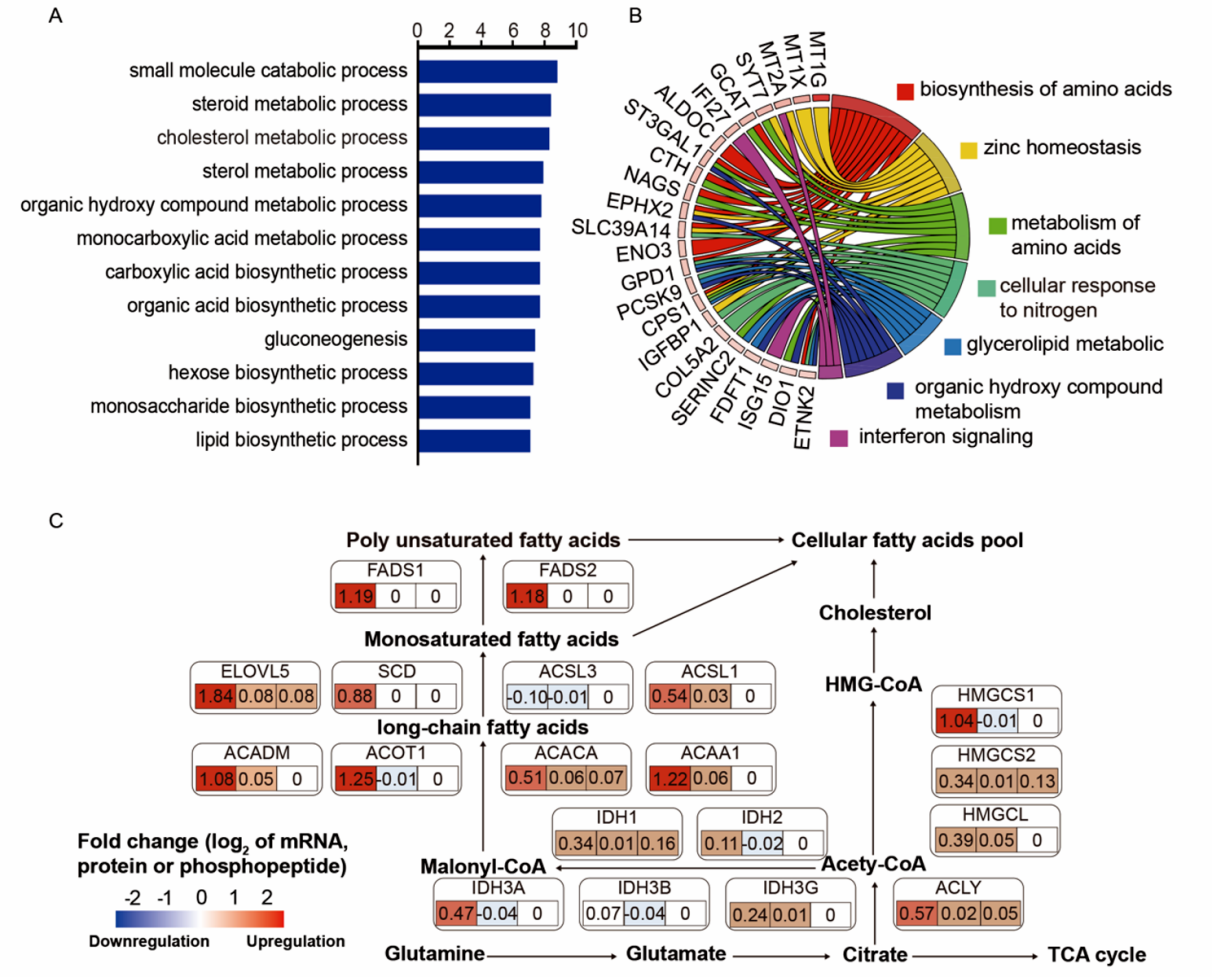
Variations in transcription between HBV^+^ and HBV^−^ HCC patients. **(A)** Gene annotation analysis for the differentially expressed genes (DEGs) between HBV^+^ and HBV^−^ HCC patients retrieved from the TCGA database (HBV^+^ HCC = 135, HBV^−^ HCC = 89).**(B)** The chord diagram indicated the top differentially expressed mRNAs (HBV^+^ vs. HBV^−^) from the Chinese human proteome program (CNHPP) and the accordingly biological functions involved. **(C)** Variations of fatty acid metabolism based on the expression of mRNAs, protein, and phospho-peptide data of CNHPP matrix (HBV-positive cancer versus adjacent tissue)

Although the comparison of 738 genes from the TCGA transcriptional matrix between 135 HBV^+^ HCC patients and 89 HBV^−^ patients revealed statistically significant differences, the PCA analysis did not exhibit clear separation associated with HBV infection, which is in line with the widely acknowledged understanding that hepatocellular carcinoma is genetically and biologically heterogeneous (Fig. S1A, Table. S6). Notably, among the most differentially expressed gene sets, the top 20 genes out of the 738 differentially expressed genes, including LGALS14, BPIFA1, MDBT1, HHATL, PCSK1, SST, CLCA1, ZNF385D-AS2, and SOX1, have previously been reported to correlate with overall survival (Table S2). Gene annotation analysis of these differentially expressed genes revealed their involvement in pathways primarily associated with the steroid metabolic process (-log10(P) = 8.4), cholesterol metabolic process (-log10(P) = 8.3), sterol metabolic process (-log10(P) = 7.9), and lipid biosynthetic process (-log10(P) = 7) (Fig. 1A). Furthermore, the analysis of protein-protein interactions identified a significant association with glycerolipid metabolism (log10P=-15.4) (Fig. S1B).

Considering the superior capability of proteomics data in reflecting gene function compared to transcriptomics data, we analyzed the proteomic characteristics of HBV-associated HCC and their adjacent tissue counterparts using the data matrix obtained from the CNHPP hepatocellular carcinoma data portal [17] (Fig. 1B). By comparing 1380 human genes from 55 metabolic pathways in the KEGG database (Table. S7), we identified 104 metabolic genes that exhibited significant differences between HBV-positive cancer versus adjacent tissue (Table. S8). The combined analysis of transcriptomic and proteomic levels of these metabolic genes highlighted a significant impact on fatty acid metabolism (as illustrated in Fig. 1C). Moreover, HBV infection disrupted the metabolism of glucose, glycerolipids, and amino acids (Fig. S1C-E, Table. S9). Notably, the metabolic enzymes involved in fatty acid metabolisms, such as fatty acid desaturase 1 (FADS1), fatty acid desaturase 2 (FADS2), acyl-CoA thioesterase 1 (ACOT1), and acetyl-CoA acyltransferase 1 (ACAA1), as well as enzymes involved in glycolysis, such as phosphofructokinase (PFKM), fructose-bisphosphate aldolase C (ALDOC), and enolase 3 (ENO3), exhibited distinct metabolic phenotypes in HBV-associated HCC compared to their adjacent tissue.

**Fig. 2:**
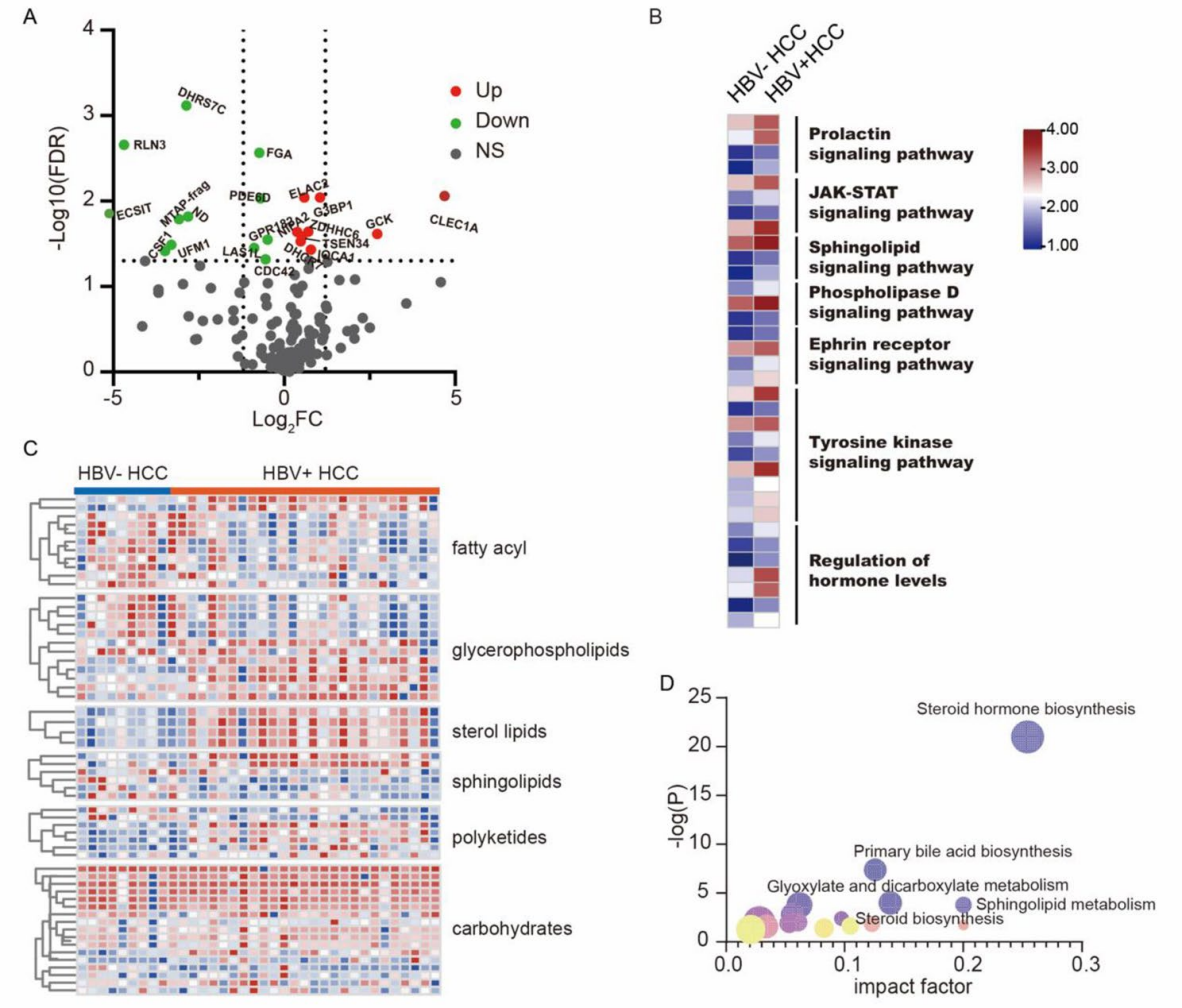
Protein profile of HCC patients with different HBV infection status. **(A)** The volcano plot illustrated the differentially expressed serum protein candidates between HBV^+^ HCC and HBV^−^ counterparts. Green dots indicate the downregulated proteins and red dots stand for the upregulated ones (FC > 1.5, *p* < 0.05, student’s t-test). **(B)** Heatmap showed the function annotation of the 74 significantly different protein candidates among HBV^−^infected HCC and HCC without HBV by meta scape, as indicated that most proteins participated in signaling transduction, such as JAK-STAT, Tyrosine kinase signaling pathway. **(C)**Metabolites heatmap showed the metabolites profile discrimination mainly attributed to lipid species, particularly fatty acyl and glycerolipids. **(D)** Metabolomic pathway analysis via MetaboAnalyst. Circle stands for the enriched pathway. The radius of the circle represented the impact of the pathway

The serum protein profile of HBV^+^, HBV^−^ HCC patients and healthy controls was determined. The serum proteomic analysis revealed distinct expression patterns in patients with HBV-associated hepatocellular carcinoma compared to healthy controls, as depicted in Supplementary (Fig. S2A). 165 protein candidates associated with hepatocellular carcinoma involved in cell membrane formation, cell adhesion, Golgi apparatus membrane, endoplasmic reticulum membrane, and exosomes were listed (Table S10, Fig. S2B). Among them, 76 proteins exhibited statistically significant differences between HBV^+^ and HBV^−^ patients (Fig. 2A, Table S11). Notably, the protein abundance was predominantly enriched in membrane signaling transduction pathways, including JAK-STAT and tyrosine kinase, as well as metabolism-related pathways such as sphingolipid signaling, phospholipase D signaling, and regulation of hormone levels (Fig. 2B). HRAS, GCK, MAPK13, SLC3A, CLEC1A and DHCR7 proteins involved in primary metabolism pathways, were ranked as the top candidate proteins when comparing HBV^+^ patients with HBV^−^ counterparts.

Furthermore, gene ontology analysis (Fig. S2C) revealed that HBV infection impacted metabolic processes and immune responses, in addition to membrane signaling and transduction. Sequential Metabolomics profiling of 36 HCC patients identified 399 statistically significant metabolites out of 802, distinguishing HBV^+^ patients from HBV^−^ patients (*P* < 0.05) (Fig. 2C). The metabolite features of 4 modes are illustrated in Fig. S3. Pathway analysis (Fig. 2D) demonstrated the involvement of differentially expressed metabolites in pathways such as primary bile acid biosynthesis (*P* = 3.41e-22), steroid hormone biosynthesis (*P* = 1.03e-21), and sphingolipid metabolism (*P =* 0.000167). These findings are consistent with bioinformatic analysis based on transcriptomic and proteomic databases, indicating disrupted lipid metabolism in HBV-associated HCC, and most lipid species were altered (Fig. 2C).

### HBV-induced sphingolipid metabolic dysfunction predicted a poor prognosis of HCC

**Fig. 3:**
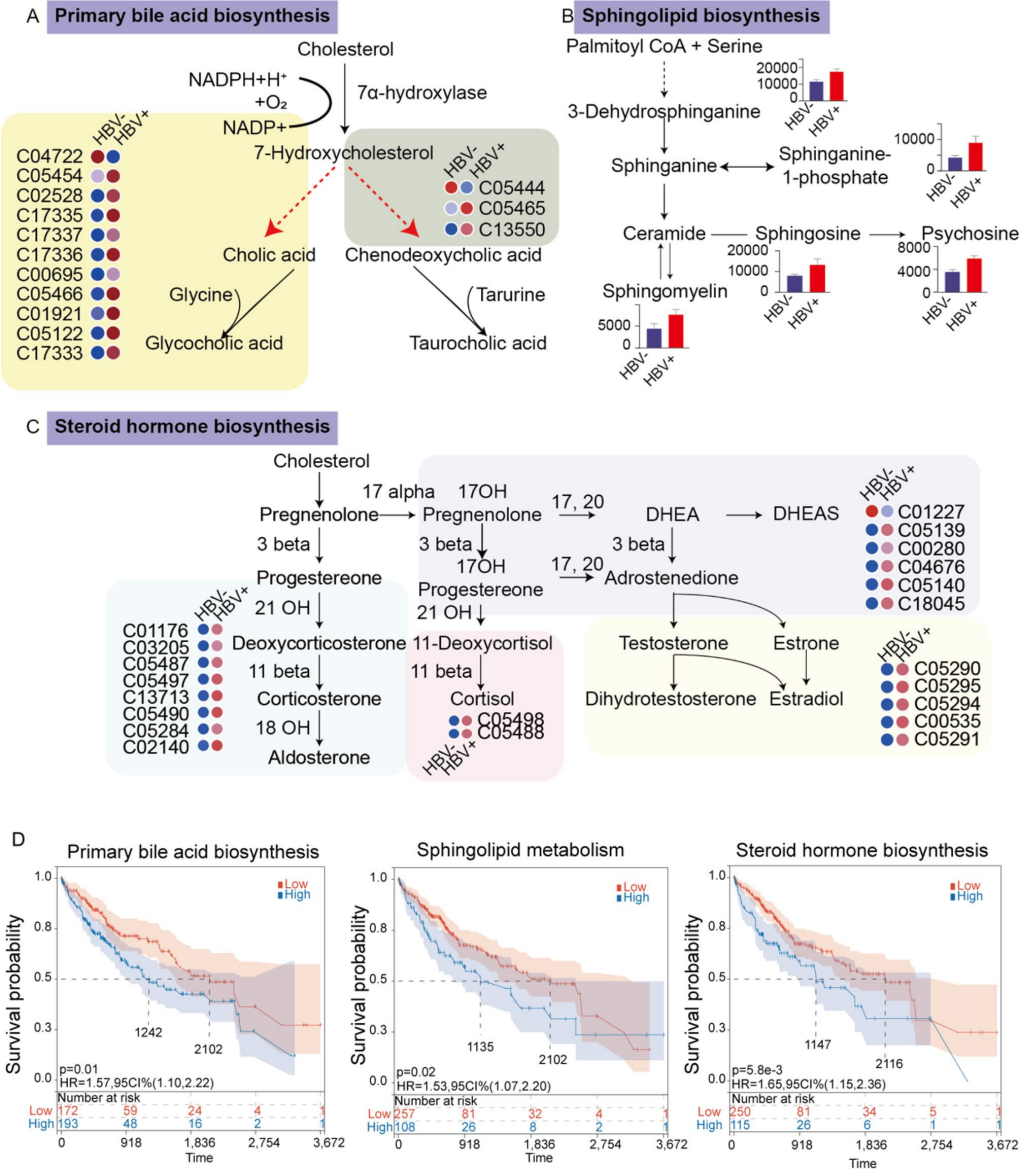
The variation of metabolic processes and their effect on overall survival. **(A)** Metabolic discrimination is involved in primary bile acid biosynthesis, in which HBV^+^ patients have higher levels than negative patients. C04722: Coprocholic acid; C05454: 5beta-Cholestane-3alpha,7alpha,12alpha-triol; C02528: Chenodeoxycholic acid; C17335: 3beta,7alpha-Dihydroxy-5-cholestenoate; C17337: 7alpha-Hydroxy-3-oxo-4-cholestenoate; C17336: 7 alpha,26-Dihydroxy-4-cholesten-3-one; C00695: Cholic acid; C05466: Chenodeoxycholic acid glycine conjugate; C01921: Glycocholic acid; C05122: Taurocholic acid; C17333: 3beta-Hydroxy-5-cholestenoic acid. **(B)** Metabolic discrimination is involved in sphingolipid biosynthesis pathways, in which HBV^+^ patients have higher levels than negative patients. **(C)** Metabolic discrimination is involved in the steroid hormone biosynthesis pathway in which HBV^+^ patients have higher levels than negative patients. C01176: 17-Hydroxyprogesterone; C03205: Deoxycorticosterone; C05487: 17alpha, 21-Dihydroxypregnenolone; C05497: 21-Deoxycortisol; C13713: Tetrahydrodeoxycorticosterone; C05490: 11-Dehydrocorticosterone; C05284: 11b-Hydroxyandrost-4-ene-3, 17-dione; C02140: Corticosterone; C05498: 11beta-Hydroxyprogesterone; C05488: Cortexolone; C01227: Dehydroepiandrosterone; C05139: 16a-Hydroxydehydroisoandrosterone; C00280:Androstenedione; C04676: Testololactone; C05140:16a-Hydroxyandrost-4-ene-3,17-dione; C18045: 7a-Hydroxydehydroepiandrosterone; C05290: 19-Hydroxyandrost-4-ene-3,17-dione; C05295: 19-Oxotestosterone; C05294: 19-Hydroxytestosterone; C00535: Testosterone; C05291: 7a-Hydroxytestosterone. **(D)** Kaplan–Meier survival analyses of high-risk and low-risk groups in the TCGA cohort associated with primary bile acid biosynthesis, sphingolipid metabolism and Steroid hormone biosynthesis

As the lipids perturbation in HBV^+^ HCC (Fig. 2C**)**, we separately compared each pathway’s metabolites changes from enrichment analysis. Figure 3A depicts the metabolites changes involved in the primary bile acid biosynthesis, particularly the metabolic process of 7-hydroxycholesterol converted to cholic acid or chenodeoxycholic acid significantly changed. Most metabolites in the HBV^+^ group were upregulated compared with the HBV^−^ counterparts. In Fig. 3B, metabolites involved in sphingolipids biosynthesis were significantly changed between HBV^+^ HCC and HBV^−^ HCC. Sphingosine-1-phosphate was markedly elevated in HBV^+^ HCC patients, indicating its potential as a biomarker for HBV^+^ HCC (Fig. 3C). During the steroid hormone biosynthesis, the production of aldosterone, cortisol, estradiol, and androsterone was enhanced obviously. Next, the correlation between pathway activity and overall survival was evaluated by GSVA. Patients with HCC retrieved from the TCGA database were stratified into two groups based on the activity level of metabolic pathways. Those with higher metabolic pathway activity were classified into the high-activity group, while those with lower activity comprised the low-activity group. The primary bile acid biosynthesis, sphingolipid metabolism and Steroid hormone biosynthesis were correlated with a poor prognosis of HCC (Fig. 3D).

### Primary bile acid and linoleic acid metabolism were altered by HBV replication

**Fig. 4:**
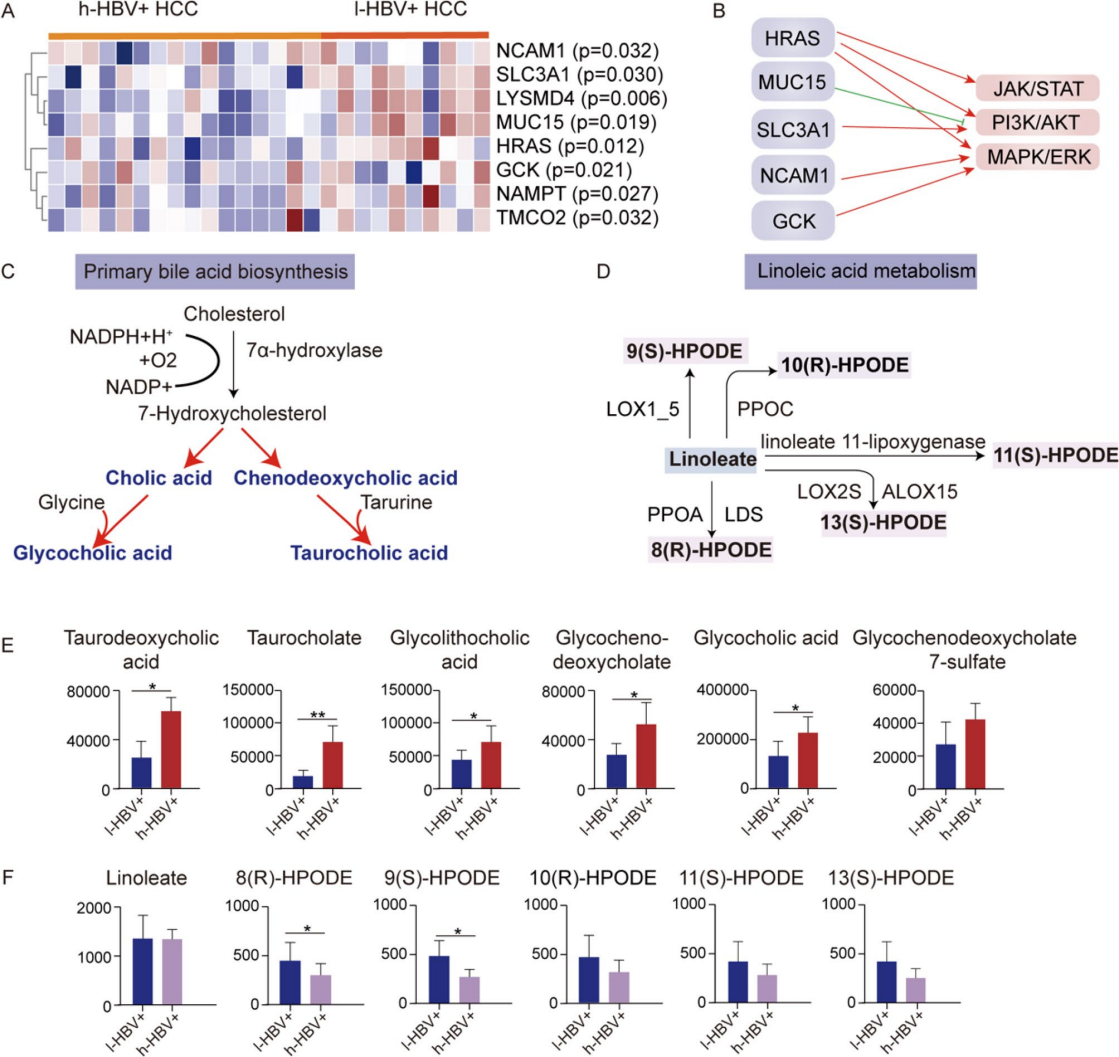
Differential lipid metabolism between HBV^−^ and HBV^+^ patients. **(A)** Heatmap showed the differential in protein abundance between h-HBV^+^ patients and l-HBV^+^ patients. **(B)** JAK/STAT, PI3K/AKT, and MAPK/ERK signaling pathways are involved in the serum proteome variations between high- and low-HBV groups. HRAS activates all three pathways, while SLC3A1 activates PI3K/AKT. MUC15 inhibits PI3K/AKT. NCAM1 and GCK also activate PI3K/AKT and MAPK/ERK. These complicated protein interactions help explain HBV^−^related pathogenesis. **(C)** The illustration showed metabolic discrimination involved in the primary bile acid biosynthesis process. **(D)** The illustration showed metabolic discrimination involved in linoleic acid metabolism. **(E)** Statistical analysis of the metabolic abundance discrimination involved in the primary bile acid biosynthesis process. **(F)** Statistical analysis of the metabolic abundance discrimination involved in linoleic acid metabolism. (*p < 0.05, **p < 0.01)

Through a comparative analysis of serum protein expression profiles between HBV^+^ patients with high virus load (h-HBV^+^) and patients with lower virus titers (l-HBV^+^), we identified 8 proteins that exhibited statistical differences (Fig. 4A). Most of these candidate proteins were involved in the membrane-related processes and metabolism, specifically HRAS in choline metabolism, MUC15 in O-glycan processing, and GCK in central carbon metabolism. Furthermore, these differential proteins are involved in the JAK/STAT, PI3K/AKT, and MAPK/ERK pathways (Fig. 4B). Those discrimination further pointed out that the HBV replication could aggravate the dysregulated metabolic environment of HCC patients, compared to patients with lower virus load. Notably, the metabolites associated with the primary bile acid biosynthesis and linoleic acid metabolism pathways exhibited virus-load-dependent alterations (Fig. 4C, D). Bile acid metabolites, such as cholic acid, glycocholic acid, and taurocholic acid, were higher in patients with a higher virus load. Conversely, the secondary metabolites involved in linoleic acid metabolism, particularly linoleate-derived metabolites including 8(R)-HPODE, 9(S)-HPODE, 10(R)-HPODE, 11(S)-HPODE, and 13(S)-HPODE, were downregulated in HBV^+^ patients with a higher virus load (Fig. 4E, F). These findings suggest that HBV replication influences bile and linoleic acid metabolism.

### Accumulation of polyunsaturated fatty acids and long-chain unsaturated fats is the primary characteristic of metabolic reprogramming in HBV-associated HCC

**Fig. 5:**
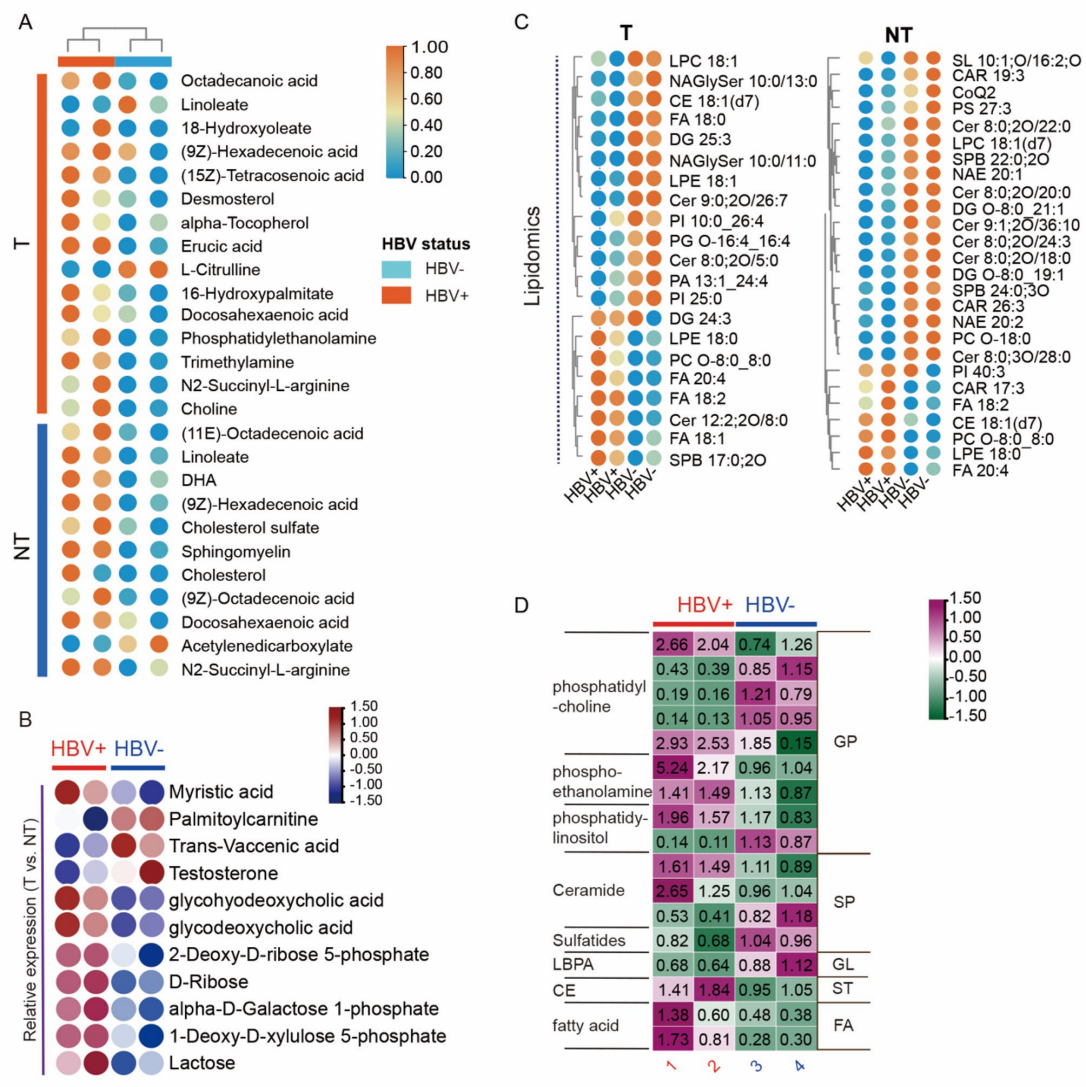
The metabolic reprogramming of HCC patients. **(A)** The metabolic features of tumor samples obtained from HBV^+^ HCC patients compared to adjacent tissue (NT) and HBV^−^ HCC patients. **(B)** The relative level of metabolic alteration of the tumor to non-tumor in HBV^+^ and HBV^−^ patients (T/NT). **(C).** Lipids alterations in HBV^+^ HCC patients compared with their counterparts, both the abundance in tumor biopsies (T) and the adjacent region (NT). **(D)** The relative level of lipid alteration of the tumor to non-tumor in HBV^+^ and HBV^−^ patients (T/NT)

Metabolic characteristics within HCC biopsies were comprehensively assessed to gain insights into the underlying metabolic alterations. The detailed list of metabolites identified is provided in Fig. 5A and Table S12. Table S3 presents the abundance of metabolites between tumor and non-tumor adjacent tissue in HBV^+^ HCC patients. Consistent with previous observations in serum metabolomics (Fig. 3A), metabolites related to bile acid metabolism, including glycohyodeoxycholic acid, glycocholate, and glycodeoxycholic acid, as well as linoleic acid, displayed consistent patterns. Within the tumor region of HBV^+^ patients, a decreasing trend in unsaturated fatty acids, such as linoleic acid and vaccenic acid, was observed compared to the non-tumor region. Moreover, the metabolite discrepancies between HBV^+^ and HBV^−^ tumors, long-chain unsaturated fatty acids, including trans-vaccenic acid and 9-trans-palmitelaidic acid, exhibited higher levels in HBV^+^ tumor tissue. But the abundance of palmitoylcarnitine was lower in HBV^+^ tumors, indicating the downregulation of palmitoylcarnitine by HBV (Table S4).

Additionally, as shown in Table S5, hepatic unsaturated fatty acid content was higher in HBV^+^ HCC non-tumor tissue compared to HBV^−^ non-tumor tissue. Specifically, 9Z,12Z-linoleic acid, α-linolenic acid, trans-vaccenic acid, oleic acid, 9-trans-palmitelaidic acid, and nervonic acid were increased in HBV^+^ hepatic tissue compared to HBV^−^ counterparts. Tissue metabolomics analysis confirmed that HBV infection induces alterations in bile acid and linoleic acid metabolism. In addition, the levels of metabolites associated with unsaturated fatty acids were higher in both tumor (T) and non-tumor (NT) adjacent tissue of HBV^+^ patients compared to those without a history of HBV infection (Fig. 5B). These results shed additional light on the metabolic dysregulation associated with HBV^+^ HCC.

Based on the identified disruptions in lipids from serum metabolites, a lipidomic methodology was utilized to examine the metabolic changes occurring in the tumor microenvironment. The glycerophospholipid lipid profiles displayed discernible dissimilarities between HBV^+^ tumors and HBV^−^ counterparts, as illustrated in Fig. 5C. Furthermore, the two groups exhibited distinct variations in the fatty acid and sphingolipids levels. The findings indicate a significant reduction in metabolites derived from phosphatidylcholinein both HBV^+^ tumors and non-tumor tissue (Fig. 5D), aligning with the observed serum patterns, indicating that there is high utilization of glycerophospholipids during viral replication. Additionally, the analysis revealed elevated levels of phosphoethanolamine and fatty acid metabolites, alongside a marked increase in ceramides within HBV^+^ tumors relative to adjacent non-tumor tissues. This differential lipidomic profile underscores the distinct metabolic reprogramming in the tumor microenvironment driven by HBV infection.

### The hyperactive MAPK/mTOR pathway are contributed to metabolic reprogramming in HBV-associated HCC

**Fig. 6:**
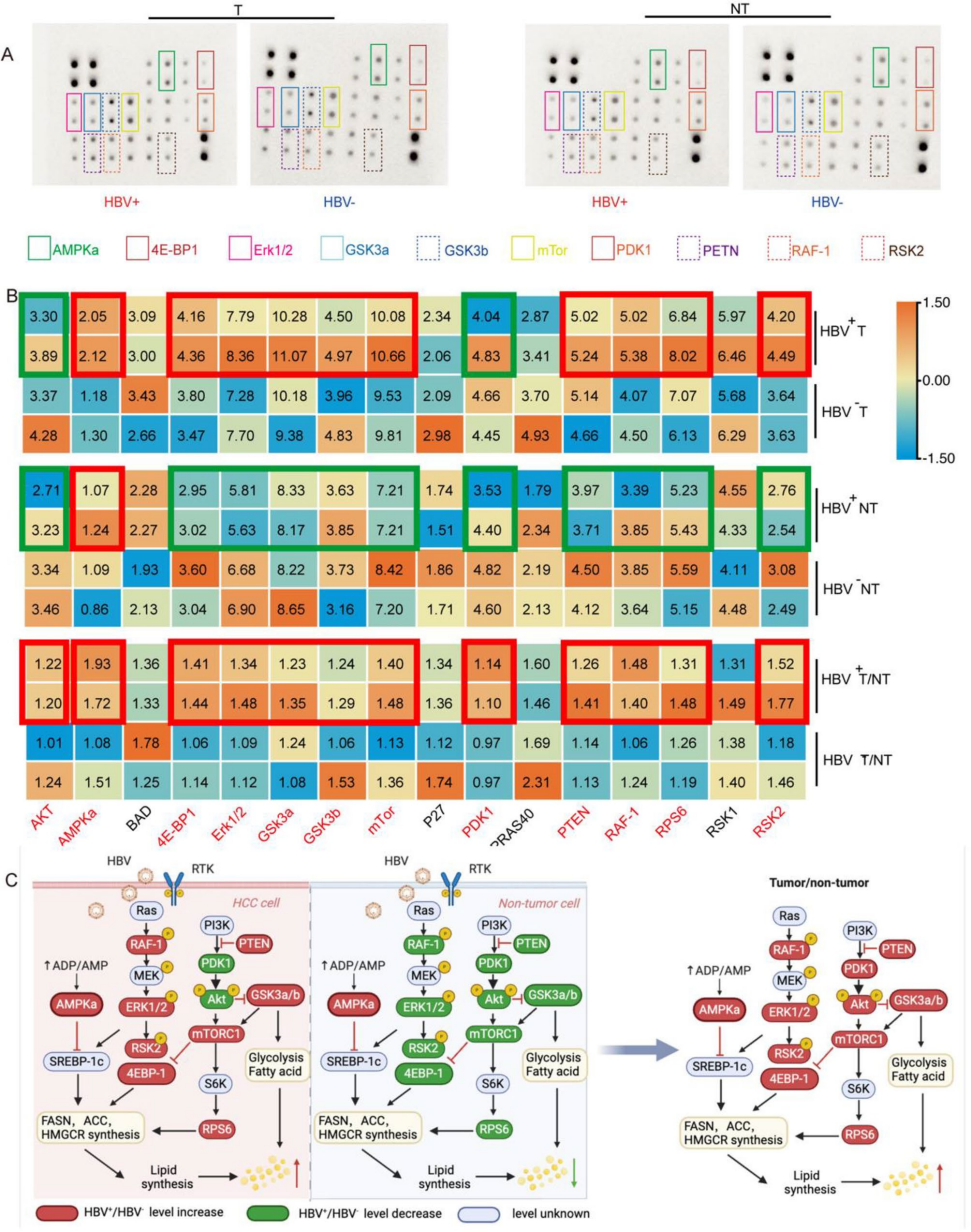
Phosphorylation antibody arrays unveil differential MAPK/mTOR protein expression in tumor and non-tumor tissues of HBV^+^ and negative HCC patients. (**A**) Image of phosphorylation antibody array to show the MAPK/Akt/mTOR signaling cascade changes of biopsies obtained from HBV^−^related HCC patients and HBV^−^ HCC patients. (**B**) Statistical analysis of phosphorylation antibody arrays; Top: the statistical results of MAPK/AKT/mTOR phosphorylation protein expression in tumor tissues of HBV positive and negative patients, respectively; Middle: Statistical results of MAPK/AKT/mTOR phosphorylation protein expression in nontumor tissues of HBV positive HCC patients and negative patients, respectively; Below: The statistical analysis of MAPK/Akt/mTOR in tumor tissues of HBV positive and negative HCC patients and the ratio to nontumor tissues. (**C**) Illustration of the significant variations of MAPK/Akt/mTOR pathway

Prior studies have indicated that the progression of HCC is intensified by fatty acids, primarily through activating the phosphatidylinositol 3-kinase pathway (PI3K) [18]. Hence, we utilized the phosphorylation antibody array method to ascertain the involvement of PI3K/Akt/mTOR in metabolic regulation. Figure 6 shows that the antibody array probed the protein expression in tumor biopsies PI3K/Akt/mTOR signaling (Fig. 6A, B). The expression levels of AMPK, 4E-BP1, Erk1/2, GSK3a, GSK3b, mTOR, PTEN, RAF-1, RPS6, and RSK2 exhibited a more remarkable fold change in HBV^+^ tumors when compared to their HBV^−^ counterparts. In contrast, the protein AKT and PDK1, a vital component of the PI3K/AKT pathway, exhibited a decreasing trend. Previous research primarily emphasized the role of the PI3K/AKT pathway in HBV infection. However, our results revealed that the PI3K/AKT pathway was not significantly up-regulated, with only the downstream mTOR pathway showing up-regulation within the entire pathway. On the other hand, the MAPK/ERK pathways demonstrated a general relative up-regulation (Fig. 6B, C), suggesting that the MAPK/mTOR axis might play a dominant role in HBV^+^ HCC, leading to a cascade of alterations, particularly in fatty acid biosynthesis and lipid metabolism, which has not been previously reported in existing studies.

We also evaluated these pathways in non-tumor tissue. We found that the MAPK pathway was downregulated in HBV-positive non-tumor tissue compared to HBV-negative non-tumor tissue. The AKT pathway was not significantly upregulated, suggesting that the upregulation of the MAPK pathway is mainly a feature of HBV-associated HCC rather than HBV itself. This phenomenon has also yet to be reported in the past. Therefore, we further compared the relative expression of these proteins by the ratio of tumor/non-tumor (T/NT). The difference in the T/NT ratio was mainly reflected in PDK1, suggesting that PDK-1 may be the most critical differential molecule in the HBV ^+^ HCC and the mechanism of HBV infection, which has not been reported in the past. Moreover, AKT seems to show the same trend as PDK1. These novel findings highlight the importance of the MAPK/mTOR axis in HBV^+^ HCC and shed light on its potential impact on lipid metabolism, revealing a previously unexplored aspect of HBV-related HCC.

## Discussion

**Fig. 7:**
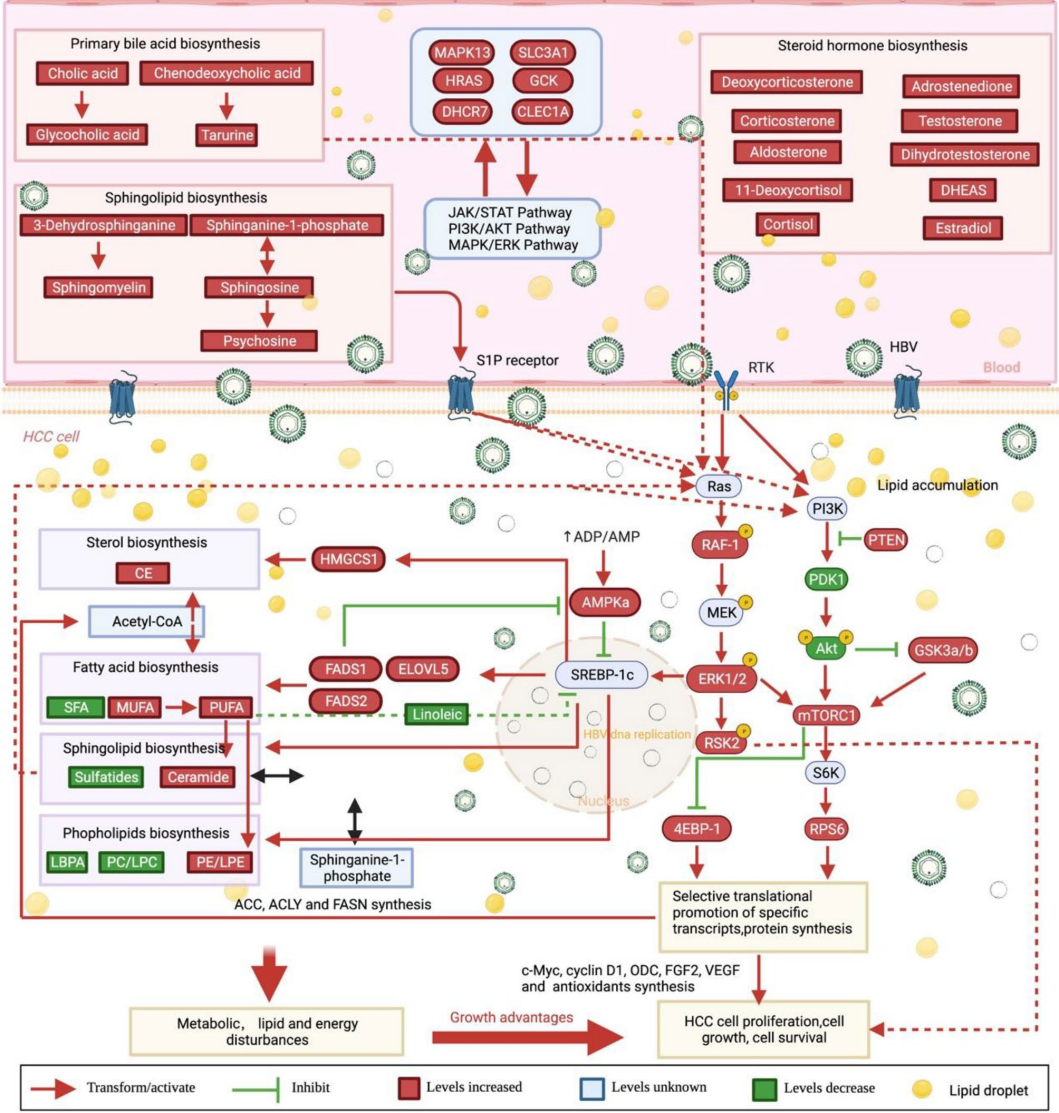
HBV^+^ patients exhibit significantly elevated levels of serum primary bile acids, sphingolipids, and steroid biosynthesis products. Notably, these primary bile acids and sphingolipid synthesis metabolites are pivotal in activating the MAPK/mTOR pathway in hepatocellular carcinoma (HCC) cells. Consequently, activating the MAPK/mTOR pathway triggers the subsequent activation of the SREBP-1 transcription factor, which fosters the biosynthesis of fatty acids, sphingolipids, and sterol. Consequently, this leads to significant reprogramming in lipid metabolism within HCC cells, further triggering the activation of the MAPK/mTOR pathway, thereby establishing a self-feeding circle driven by metabolites. Ultimately, this process may fuel the malignant biological behaviors of increased proliferation, invasion, metastasis, and other malignancy-associated characteristics

In this study, we conducted a comprehensive analysis of transcriptomic and proteomic data obtained from TCGA and CNHPP databases. Additionally, we investigated the proteome, metabolome, and lipidome of our hepatocellular carcinoma cohort. By integrating genomics, proteomics, metabolomics, and lipidomics, we employed a multi-omics approach to elucidate the metabolic reprogramming and underlying mechanisms in HBV-associated HCC (Scheme 1). Our findings highlight the significance of primary bile acid anabolic metabolites and sphingolipid metabolites in serum, which play critical roles in activating metabolic disorders in HBV^+^ HCC. Specifically, bile acid and sphingolipid metabolites activate the hepatocellular carcinoma MAPK/mTOR pathway, subsequently leading to lipid metabolism disorders in HCC – a phenomenon not previously reported. Moreover, we observed that the perturbation of linoleic acid metabolism and primary bile acid metabolism signaling pathways occurred in a viral load-dependent manner. Our tissue metabolomic analysis partly confirmed the serum metabolome results, revealing distinct metabolic profiles between HBV^+^HCC and HBV^−^HCC. Notably, significantly elevated levels of polyunsaturated fatty acids were evident in hepatitis B-associated hepatocellular carcinoma. In exploring central energy metabolism signaling pathways using protein arrays, we identified substantial changes in the MAPK/mTOR signaling cascade. Remarkably, the MAPK pathway played a dominant role, contrasting with the previously identified AKT pathway, as depicted in Fig. 7. Our multi-omics approach sheds light on the intricate interplay among viral infection, metabolic reprogramming, and signaling pathways in HBV-associated hepatocellular carcinoma (HCC). These findings not only elucidate the underlying mechanisms driving HBV-associated HCC but also unveil potential treatment avenues for this challenging malignancy.

### Firstly, a distinct metabolic signature orchestrated by HBV confers a survival advantage in HCC tumor cells

Hence, HBV^+^ HCC necessitates specific therapeutic interventions. Despite recent advancements in sequencing-based studies that have provided comprehensive insights into the genetic landscape of HBV^−^related HCC [19], the precise impact of the virus on cancer progression remains elusive. To address this problem, our study leveraged the Cancer Genome Atlas (TCGA) transcriptomic data to identify 738 differentially expressed genes (DEGs) between HBV^+^ and HBV^−^ HCC patients. Among these DEGs, up-regulated genes in HBV^+^ HCC, including LGALS14, BPIFA1, MDBT1, and HHATL, were associated with poorer prognosis [20–23]. Furthermore, pathway enrichment analysis of these differential genes revealed significant distinctions between HBV^+^ and HBV^−^ HCC, primarily in amino acid metabolism, cholesterol metabolism (-log10(P) = 8.3), and lipid biosynthesis (-log10(P) = 7). The enhanced amino acid metabolism, cholesterol metabolism, and fatty acid biosynthesis contribute to a constant energy supply for cancer cells, fulfilling the energy demands of malignant biological behaviors, such as proliferation and invasion [24–26].

### HBV-associated HCC cells exploit the self-feeding mechanism of bile acids and sphingolipids to promote their malignant biological behavior

Previous studies have consistently reported that HCC patients had higher bile acid levels, which correlate with HCC development [27, 28]. In line with these findings, our results showed increased activity in the primary bile acid biosynthesis in the serum of HBV^+^ HCC patients compared to the HBV^−^ counterpart. Moreover, metabolites involved in primary bile acid production increased in HBV^+^ patients with increasing virus loads. Analysis of the metabolic profiles obtained from HBV^+^ HCC biopsies also revealed an abundance of metabolites associated with the bile acid biosynthesis process, including cholic acid, chenodeoxycholic acid, and related intermediates. Several studies have consistently demonstrated that primary bile acid synthesis products can activate the MAPK/ERK pathway [29–31]. Sphingolipids are known for their role in cell membrane formation and contribution to membrane flexibility and trafficking [32]. Increasing serum sphingolipids in HBV infection illustrated a cancer progression [33, 34]. In our work, HBV^+^ HCC upregulated sphingolipids in serum and tumor tissue. (Figures 3B and 5A). The lipid profile exhibited ceramide and phingosine-1-phosphate disruption (Fig. 5D). Among them, sphingosine-1-phosphate (S1P) plays a significant role, as it can enter cells via the S1P receptor, subsequently activating the RAS/MAPK and PI3K/AKT pathways [35–37]. Notably, our tumor tissue metabolic and lipid profile analysis revealed that excessive activation of the MAPK/ERK pathway in HCC cells could further augment the primary bile acid synthesis and sphingolipids metabolism in turn. Thus, it is reasonable to speculate that the excessive accumulation of bile acids and sphingolipids in HBV-associated HCC patients triggers lipid metabolism reprogramming via the activation of the MAPK/mTOR pathway in HCC cells. Moreover, the metabolites of bile acids and sphingolipids can further activate the MAPK/mTOR pathway, establishing a self-feeding pattern that reinforces the malignant biological behavior of HCC.

These findings highlight the potential of targeting bile acid and sphingolipids metabolism as a promising therapeutic strategy for HBV^+^ HCC patients.

### HBV-associated HCC has distinct metabolic features, especially in accumulating unsaturated fatty acids

Metabolic changes induced by HBV, such as upregulation of hexosamine and phosphatidylcholine, have been observed in previous research [38]. However, the mechanisms behind liver injury, HCC malignant progression in HBV^−^infected patients, and identifying metabolic pathways as cancer therapeutic targets are poorly known. Our study has unveiled that Fatty acyl, glycerophospholipids, sterol lipids, and sphingolipids were distinct in HBV^+^ HCC patients. Lipid metabolites were accumulated in primary bile acids and sphingolipids. In addition, HBV^+^ HCC biopsy has higher glycerophospholipids, including lyso-phosphocholine (18:2), compared to either the non-tumor tissue from the same patients or the HBV^−^ HCC biopsy. Unsaturated fatty acids may cause carcinogenesis and interact with immunological regulators, predicting a poor prognosis for HCC [39, 40]. In HBV^+^ patient biopsies, the unsaturated fatty acids that are essential for cancer progression, such as 9z,12z-linoleic acid, α-linolenic acid, trans-vaccenic acid, oleic acid, 9-trans-palmitelaidic acid, and nervonic acid [41], are higher than HBV^−^ counterpart. Our findings highlight the altered lipid profile observed in HBV^+^ HCC patients, particularly the accumulation of unsaturated fatty acids. Understanding these metabolic alterations may help design tailored HBV^−^related HCC treatments.

### MAPK/mTOR resulted in lipogenesis contributing to HBV-associated HCC’s metabolic alterations more important than the previously widely focused AKT pathway

Consistent with the AKT pathway, MAPK/mTOR signaling cascade is also pivotal in growth, proliferation, and cell survival [42, 43]. Our antibody array analysis revealed hyperactivation of the MAPK/mTOR pathway, particularly the upregulation of 4EBP1, which is involved in the mTORC1-dependent glycolytic pathway. The phosphorylation state 4EBP-1 synthesizes specific proteins, such as FASN, ACC, and HMGCR, which enhances lipid synthesis [44–46]. Consistent with these findings, our result demonstrated that the MAPK/mTOR pathway regulates lipid metabolism by modulating key proteins, including SREBP-1, 4EBP-1, and RPS6, primarily by ERK1/2. These interactions subsequently govern the functional proteins involved in lipid production and contribute to the reprogramming of lipid metabolism. This intricate mutual crosstalk signaling axis had not been reported in previous studies, and our result further underscored the MAPK/mTOR potential as a therapeutic target for HBV^−^related HCC patients.

## Conclusion

By integrating transcriptomic and proteomic data from various databases with our comprehensive analysis of proteins and metabolites, we aimed to elucidate the effects of HBV on HCC beyond the established mechanisms of DNA insertion, epigenetic regulation, and metabolic reprogramming. Our findings collectively underscore the significant role of HBV in hepatocarcinogenesis and tumor progression, with a particular emphasis on lipid metabolism, including steroid hormone biosynthesis, primary bile acid metabolism, and linoleic acid metabolism. HBV infection results in the excessive accumulation of bile acids and sphingolipids in the serum, leading to the activation of the MAPK/mTOR pathway in HBV^+^ hepatoma cells and subsequent reprogramming of lipid metabolism in hepatocellular carcinoma. Furthermore, these metabolites further reinforce the activation of the MAPK/mTOR pathway, forming a self-feeding vicious circle that creates a dominant pro-proliferation environment for HCC cells, posing significant challenges in the treatment of HBV-related HCC. In addition to the consistent metabolic profile observed in biopsies from HBV^+^ HCC patients, there were notable alterations in lipid composition, especially in unsaturated fatty acids. These changes in lipid metabolism can be attributed to the hyperactivation of the MAPK/mTOR signaling cascade. Our study highlights the intricate interplay between HBV infection, lipid metabolism, and HCC progression. These findings hold important implications, suggesting the necessity to distinguish between HBV-negative and HBV-positive HCC patients. Furthermore, the appropriate adjuvant use of the MAPK pathway or mTOR inhibitors could improve the prognosis of HBV-positive patients.

### Statistical analysis

Statistical analysis of qualitative and quantitative differences between subgroups was performed using appropriate methods, including χ2 test or Fisher’s exact test for categorical parameters and Student’s t-test or Mann-Whitney U test for continuous variables. Unsupervised clustering and heatmap generation based on Pearson correlation and Ward method linkage analysis.

### Electronic supplementary material

Below is the link to the electronic supplementary material.

Supplementary Material 1

## Data Availability

No datasets were generated or analysed during the current study.

## References

[1] Chagas AL, Felga GEG, Diniz MA, Silva RF, Mattos AA, Silva R (2019). Hepatocellular carcinoma recurrence after liver transplantation in a Brazilian multicenter study: clinical profile and prognostic factors of survival. Eur J Gastroenterol Hepatol.

[2] Vogel A, Rimassa L, Sun HC, Abou-Alfa GK, El-Khoueiry A, Pinato DJ (2021). Comparative efficacy of Atezolizumab plus Bevacizumab and Other Treatment options for patients with Unresectable Hepatocellular Carcinoma: A Network Meta-Analysis. Liver Cancer.

[3] El-Serag HB (2012). Epidemiology of viral hepatitis and hepatocellular carcinoma. Gastroenterology.

[4] Sangineto M, Villani R, Cavallone F, Romano A, Loizzi D, Serviddio G. Lipid metabolism in Development and Progression of Hepatocellular Carcinoma. Cancers (Basel). 2020;12. 10.3390/cancers12061419 PMC7352397 32486341

[5] Bar-Yishay I, Shaul Y, Shlomai A (2011). Hepatocyte metabolic signalling pathways and regulation of hepatitis B virus expression. Liver Int.

[6] Arbuthnot P, Kew M (2001). Hepatitis B virus and hepatocellular carcinoma. Int J Exp Pathol.

[7] Magon KL, Parish JL (2021). From infection to cancer: how DNA tumour viruses alter host cell central carbon and lipid metabolism. Open Biol.

[8] Xue X, Liao W, Xing Y (2020). Comparison of clinical features and outcomes between HBV-related and non-B non-C hepatocellular carcinoma. Infect Agents Cancer.

[9] Levrero M, Zucman-Rossi J (2016). Mechanisms of HBV-induced hepatocellular carcinoma. J Hepatol.

[10] Zhang C, Huang C, Sui X, Zhong X, Yang W, Hu X (2019). Association between gene methylation and HBV infection in hepatocellular carcinoma: a meta-analysis. J Cancer.

[11] Chen W, Jiang J, Gong L, Shu Z, Xiang D, Zhang X (2021). Hepatitis B virus P protein initiates glycolytic bypass in HBV-related hepatocellular carcinoma via a FOXO3/miRNA-30b-5p/MINPP1 axis. J Experimental Clin Cancer Res.

[12] Gao Q, Zhu H, Dong L, Shi W, Chen R, Song Z et al. Integrated Proteogenomic characterization of HBV-Related Hepatocellular Carcinoma. Cell. 2019;179:561 – 77.e22. 10.1016/j.cell.2019.08.052 31585088

[13] Jeong JS, Jiang L, Albino E, Marrero J, Rho HS, Hu J (2012). Rapid identification of monospecific monoclonal antibodies using a human proteome microarray. Mol Cell Proteom.

[14] Bolstad BM, Irizarry RA, Astrand M, Speed TP (2003). A comparison of normalization methods for high density oligonucleotide array data based on variance and bias. Bioinformatics.

[15] Want EJ, Masson P, Michopoulos F, Wilson ID, Theodoridis G, Plumb RS (2013). Global metabolic profiling of animal and human tissues via UPLC-MS. Nat Protoc.

[16] Xuan Q, Hu C, Yu D, Wang L, Zhou Y, Zhao X (2018). Development of a high Coverage Pseudotargeted Lipidomics Method based on Ultra-high Performance Liquid Chromatography-Mass Spectrometry. Anal Chem.

[17] Jiang Y, Sun A, Zhao Y, Ying W, Sun H, Yang X (2019). Proteomics identifies new therapeutic targets of early-stage hepatocellular carcinoma. Nature.

[18] Sun EJ, Wankell M, Palamuthusingam P, McFarlane C, Hebbard L. Targeting the PI3K/Akt/mTOR pathway in Hepatocellular Carcinoma. Biomedicines. 2021;9. 10.3390/biomedicines9111639 PMC8615614 34829868

[19] Gao Q, Zhu H, Dong L, Shi W, Chen R, Song Z (2019). Integrated Proteogenomic characterization of HBV-Related Hepatocellular Carcinoma. Cell.

[20] Cho Y, Lee YB, Lee JH, Lee DH, Cho EJ, Yu SJ (2016). Modified AS1411 aptamer suppresses Hepatocellular Carcinoma by Up-Regulating Galectin-14. PLoS ONE.

[21] Sentani K, Oue N, Sakamoto N, Arihiro K, Aoyagi K, Sasaki H (2008). Gene expression profiling with microarray and SAGE identifies PLUNC as a marker for hepatoid adenocarcinoma of the stomach. Mod Pathol.

[22] Frau M, Simile MM, Tomasi ML, Demartis MI, Daino L, Seddaiu MA (2012). An expression signature of phenotypic resistance to hepatocellular carcinoma identified by cross-species gene expression analysis. Cell Oncol (Dordr).

[23] Liu Y, Wang H, Yang W, Qian Y (2020). Prediction of specific subtypes and common markers of Non-small Cell Lung Cancer based on competing endogenous RNA network. Med Sci Monit.

[24] Satriano L, Lewinska M, Rodrigues PM, Banales JM, Andersen JB (2019). Metabolic rearrangements in primary liver cancers: cause and consequences. Nat Rev Gastroenterol Hepatol.

[25] Pham DV, Park PH. Tumor metabolic reprogramming by Adipokines as a critical driver of obesity-Associated Cancer Progression. Int J Mol Sci. 2021;22. 10.3390/ijms22031444 PMC7867092 33535537

[26] Luo X, Cheng C, Tan Z, Li N, Tang M, Yang L (2017). Emerging roles of lipid metabolism in cancer metastasis. Mol Cancer.

[27] Oehler N, Volz T, Bhadra OD, Kah J, Allweiss L, Giersch K (2014). Binding of hepatitis B virus to its cellular receptor alters the expression profile of genes of bile acid metabolism. Hepatology.

[28] Chen T, Xie G, Wang X, Fan J, Qiu Y, Zheng X (2011). Serum and urine metabolite profiling reveals potential biomarkers of human hepatocellular carcinoma. Mol Cell Proteom.

[29] Xu Z, Tavares-Sanchez OL, Li Q, Fernando J, Rodriguez CM, Studer EJ (2007). Activation of bile acid biosynthesis by the p38 mitogen-activated protein kinase (MAPK): hepatocyte nuclear factor-4alpha phosphorylation by the p38 MAPK is required for cholesterol 7alpha-hydroxylase expression. J Biol Chem.

[30] Chiang JY (2009). Bile acids: regulation of synthesis. J Lipid Res.

[31] Matsumoto E, Hatanaka M, Bohgaki M, Maeda S (2006). PKC pathway and ERK/MAPK pathway are required for induction of cyclin D1 and p21Waf1 during 12-o-tetradecanoylphorbol 13-acetate-induced differentiation of myeloleukemia cells. Kobe J Med Sci.

[32] Merrill AH (2011). Jr. Sphingolipid and glycosphingolipid metabolic pathways in the era of sphingolipidomics. Chem Rev.

[33] Miura K, Nagahashi M, Prasoon P, Hirose Y, Kobayashi T, Sakata J (2021). Dysregulation of sphingolipid metabolic enzymes leads to high levels of sphingosine-1-phosphate and ceramide in human hepatocellular carcinoma. Hepatol Res.

[34] Wu T, Zheng X, Yang M, Zhao A, Li M, Chen T (2017). Serum lipid alterations identified in chronic hepatitis B, hepatitis B virus-associated cirrhosis and carcinoma patients. Sci Rep.

[35] Rutherford C, Childs S, Ohotski J, McGlynn L, Riddick M, MacFarlane S (2013). Regulation of cell survival by sphingosine-1-phosphate receptor S1P1 via reciprocal ERK-dependent suppression of Bim and PI-3-kinase/protein kinase C-mediated upregulation of Mcl-1. Cell Death Dis.

[36] Park JH, Park KK, Choe JY, Jang KM (2021). Identification of sphingosine 1-phosphate level and MAPK/ERK signaling in pancreatic β cells. Ann Pediatr Endocrinol Metab.

[37] Igarashi J, Bernier SG, Michel T (2001). Sphingosine 1-phosphate and activation of endothelial nitric-oxide synthase. Differential regulation of akt and MAP kinase pathways by EDG and bradykinin receptors in vascular endothelial cells. J Biol Chem.

[38] Li H, Zhu W, Zhang L, Lei H, Wu X, Guo L (2015). The metabolic responses to hepatitis B virus infection shed new light on pathogenesis and targets for treatment. Sci Rep.

[39] Qin XY, Su T, Yu W, Kojima S (2020). Lipid desaturation-associated endoplasmic reticulum stress regulates MYCN gene expression in hepatocellular carcinoma cells. Cell Death Dis.

[40] Chen D, Zhang Y, Wang W, Chen H, Ling T, Yang R (2021). Identification and characterization of Robust Hepatocellular Carcinoma Prognostic subtypes based on an integrative metabolite-protein Interaction Network. Adv Sci (Weinh).

[41] Ackerman D, Simon MC (2014). Hypoxia, lipids, and cancer: surviving the harsh tumor microenvironment. Trends Cell Biol.

[42] Pollicino T, Cacciola I, Saffioti F, Raimondo G (2014). Hepatitis B virus PreS/S gene variants: pathobiology and clinical implications. J Hepatol.

[43] Li L, Zhao GD, Shi Z, Qi LL, Zhou LY, Fu ZX (2016). The Ras/Raf/MEK/ERK signaling pathway and its role in the occurrence and development of HCC. Oncol Lett.

[44] Guo Z, Cheng X, Feng X, Zhao K, Zhang M, Yao R (2019). The mTORC1/4EBP1/PPARγ Axis mediates insulin-Induced Lipogenesis by regulating lipogenic gene expression in bovine mammary epithelial cells. J Agric Food Chem.

[45] Musa J, Orth MF, Dallmayer M, Baldauf M, Pardo C, Rotblat B (2016). Eukaryotic initiation factor 4E-binding protein 1 (4E-BP1): a master regulator of mRNA translation involved in tumorigenesis. Oncogene.

[46] Shin S, Wolgamott L, Tcherkezian J, Vallabhapurapu S, Yu Y, Roux PP (2014). Glycogen synthase kinase-3beta positively regulates protein synthesis and cell proliferation through the regulation of translation initiation factor 4E-binding protein 1. Oncogene.

