# Supplementary material for: Metabolic self-feeding in HBV-associated hepatocarcinoma centered on feedback between circulation lipids and the cellular MAPK/mTOR axis
Source: Cell Commun Signal. 2024 May 21;22:280. doi: 10.1186/s12964-024-01619-5 (PMC11106961; doi:10.1186/s12964-024-01619-5)
Supplement: Supplementary file 1 — Supplementary Material 1 [file 12964_2024_1619_MOESM1_ESM.docx]

**Metabolic Self-feeding in HBV-associated hepatocarcinoma centered on feedback between circulation lipids and the cellular MAPK/mTOR axis**

*Running title: Metabolic Self-feeding in HBV-associated HCC*

Ying Zhu ^a, c#^, Yingke Zhao ^a, f#^, Zhouyu Ning ^a^, Yong Deng ^b,d,e^, Bing Li ^b,d,e^, Yun Sun^b, d, e^*, Zhiqiang Meng ^a, c^*

a Minimally invasive therapy center, Fudan University Shanghai Cancer Center, Shanghai 200032, China

b Department of Research and Development, Shanghai Proton and Heavy Ion Center, Fudan University Shanghai Cancer Center, Shanghai 201321, China

c Cancer Institute, Fudan University Shanghai Cancer Center, Shanghai 200032, China

d Shanghai Key Laboratory of radiation oncology (20dz2261000), Shanghai 201321, China

e Shanghai Engineering Research Center of Proton and Heavy Ion Radiation Therapy, Shanghai 201321, China.

f Eye Institute, Eye and ENT Hospital, College of Medicine, Fudan University, Shanghai,China.

***** Corresponding author:

Zhiqiang Meng, Minimally Invasive Therapy Center, Shanghai Cancer Center, Fudan University, Shanghai 200032, China; E-mail: mengshca@fudan.edu.cn.

Yun Sun, Department of Research and Development and Department of Nuclear Medicine, Shanghai Proton and Heavy Ion Center, Fudan University Shanghai Cancer Center, Shanghai 201321, China; E-mail: [yun.sun@sphic.org.cn](mailto:yun.sun@sphic.org.cn).

# Ying Zhu and Yingke Zhao contributed equally to this work

**Supporting Figures**


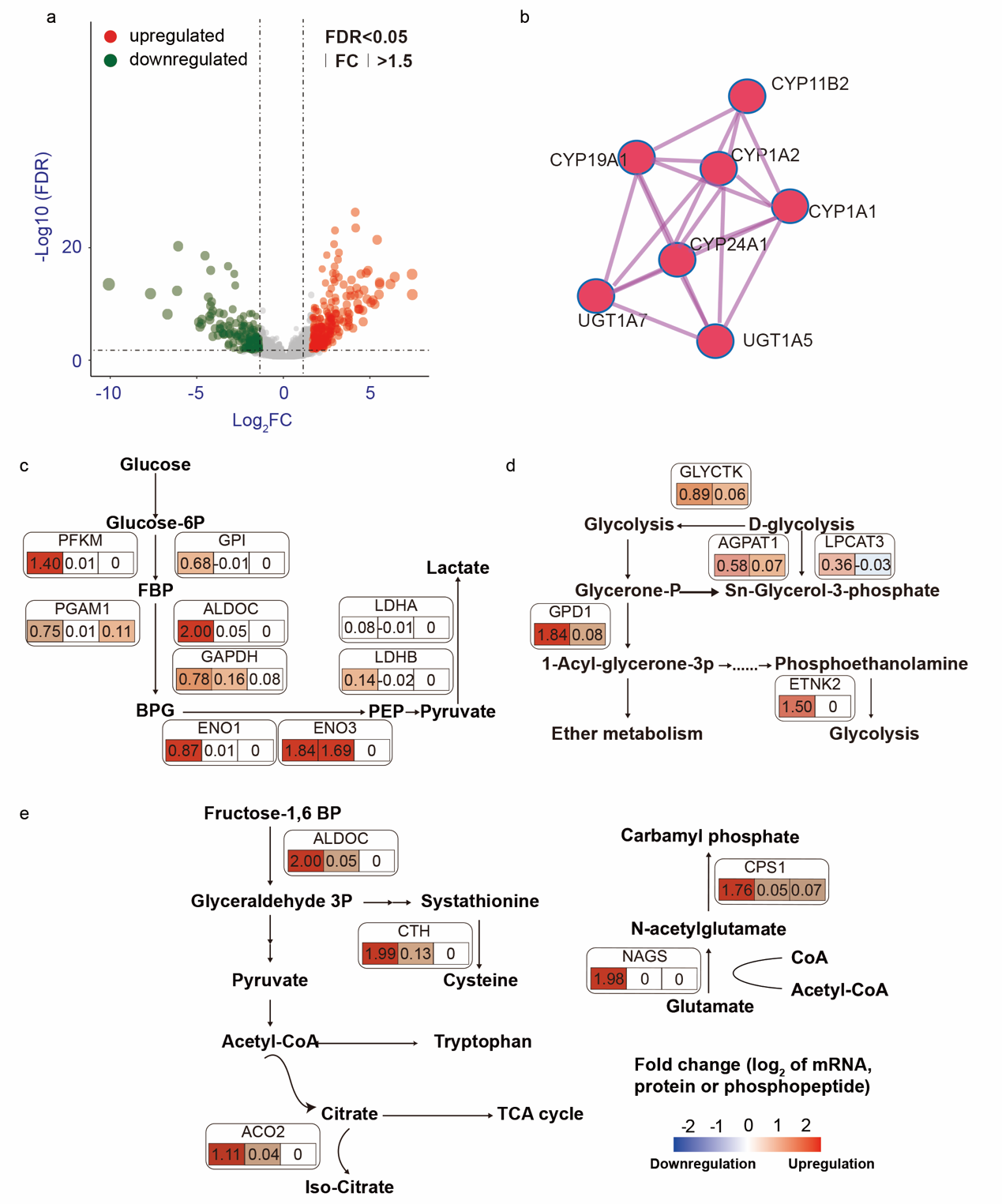


**Figure S1** **(A)** The volcano plot showed statistically significant differences in gene from the TCGA transcriptional matrix. **(B)** The analysis of protein-protein interactions identified a significant association with glycerolipid metabolism (log10P=-15.4). **(C-E)** The proteomic characteristics of HBV-associated HCC HBV infection. (The disrupted metabolism of glucose, glycerolipids, and amino acids, separately)


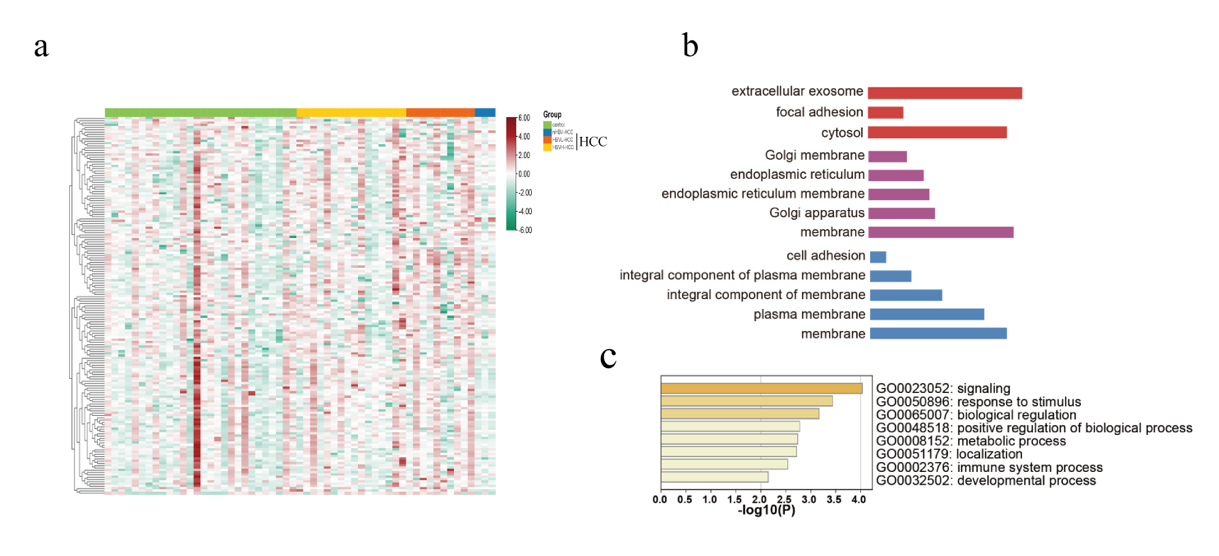


**Figure S2** **(A)** The heatmap shows HuProt™ array-identified differential expression profiles of 165 proteins in 28 healthy controls and 29 HCC patients. **(B)** The KEGG enrichment analysis of HBV-positive and HBV-negative HCC patients. **(C)** The ontology analysis of HBV-positive and HBV-negative HCC patients.


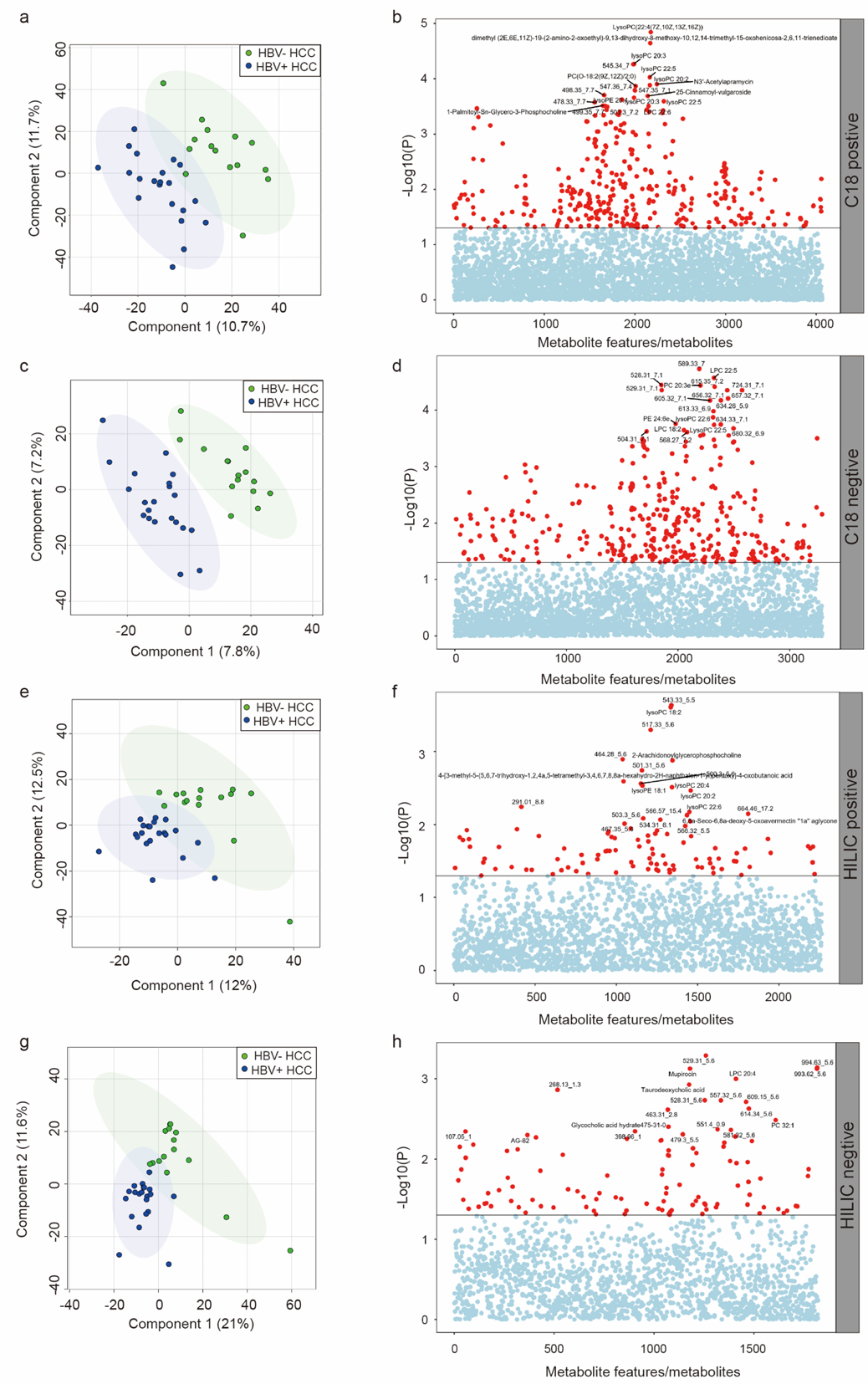


**Figure S3** The PCA analysis of HBV-positive and HBV-negative HCC patients of 4 modes**.**

**Supporting Tables**

Table S1. Clinical characteristics of recruited subjects.

| **Characteristics** | **Health Control** | **HBV^+^ HCC** | **HBV^-^ HCC** |
| --- | --- | --- | --- |
| **n** | 28 | 49 | 15 |
| **age** | 45.04±12.06 | 56.82±11.70 | 53.60±9.78 |
| **Sex (male)** | 28 | 49 | 15 |
| **AFP** | - | 226±34.46 | 116.1±58.36 |
| **HBV** | - | 49 | - |
| **AST** | - | 69.28±11.03 | 28.6±3.704 |
| **ALT** | - | 86.27±9.627 | 38.31±6.110 |
| **CA-199** | - | 66.56±24.58 | 132.4±63.94 |

**Table S2. Top 20 upregulated and downregulated genes.**

| **Upregulated** | | | **Downregulated** | | |
| --- | --- | --- | --- | --- | --- |
| **Gene names** | **logFC** | **p-value** | **Gene names** | **logFC** | **p-value** |
| **MT1B** | 10.2328 | 2.62E-16 | **PCSK1** | -7.53782 | 3.68E-62 |
| **GPR50** | 7.780407 | 1.64E-14 | **SST** | -7.33738 | 2.78E-14 |
| **TNP1** | 6.892576 | 1.18E-10 | **CLCA1** | -7.25852 | 2.92E-18 |
| **STRC** | 6.230527 | 6.87E-24 | **AC152010.1** | -6.30304 | 1.67E-17 |
| **TRARG1** | 6.143218 | 7.01E-15 | **PHOX2A** | -6.24551 | 1.24E-17 |
| **RNU6ATAC** | 5.890879 | 8.29E-22 | **NELL1** | -6.00629 | 2.86E-16 |
| **RNU6-554P** | 5.266137 | 1.84E-11 | **ZNF385D-AS2** | -5.55186 | 9.45E-12 |
| **AC009312.1** | 5.045021 | 7.25E-10 | **PAK5** | -5.43078 | 9.87E-17 |
| **LGALS14** | 5.022113 | 3.75E-09 | **SOX1** | -5.41698 | 2.14E-16 |
| **LINC02054** | 4.988967 | 1.92E-14 | **MOS** | -5.37088 | 6.73E-14 |
| **BPIFA1** | 4.969714 | 4.54E-08 | **MUC2** | -5.22148 | 9.12E-25 |
| **EDDM3B** | 4.891035 | 6.70E-09 | **FADS2B** | -5.1755 | 1.73E-13 |
| **PNLIP** | 4.888525 | 9.37E-10 | **FGF4** | -5.09179 | 2.01E-12 |
| **AC009646.2** | 4.738481 | 1.09E-06 | **METTL21C** | -5.00554 | 5.92E-13 |
| **KC877982.1** | 4.709765 | 5.33E-07 | **DLGAP1-AS5** | -5.00535 | 3.68E-16 |
| **MT1CP** | 4.704529 | 1.25E-15 | **AL136140.1** | -4.9734 | 1.28E-11 |
| **AC021393.1** | 4.699334 | 2.62E-08 | **AC109780.1** | -4.89645 | 1.30E-17 |
| **C16orf89** | 4.528714 | 1.70E-21 | **DMRTB1** | -4.88103 | 8.39E-13 |
| **TPRKBP1** | 4.496049 | 1.03E-09 | **AC104793.1** | -4.86705 | 4.86E-14 |
| **AC243972.2** | 4.362346 | 1.74E-10 | **LINC02583** | -4.80742 | 4.33E-12 |

**Tab S3. Metabolic reprogramming in HBV^+^ HCC patients (T/NT，n=2)**

| **Metabolites** | **Fold change** | | |  |
| --- | --- | --- | --- | --- |
|  | **Sample1 Sample2** | | |  |
| **Myristic acid** | | 1.22 | 1.14 | |
| **9Z,12Z-Linoleic acid** | | 0.91 | 0.62 | |
| **Trans-Vaccenic acid** | | 0.56 | 0.72 | |
| **LPC 18:2** | | 1.29 | 1.75 | |
| **Palmitoylcarnitine** | | 0.75 | 0.27 | |
| **Cholic acid** | | 0.80 | 0.73 | |
| **Glycohyodeoxycholic acid** | | 4.08 | 3.01 | |
| **Glycocholate** | | 3.51 | 1.28 | |
| **Glycodeoxycholic acid** | | 3.77 | 2.82 | |

**Tab S4. Metabolic differential between tumor tissue of HBV^+^ HCC patients and HBV^-^ negative counterparts (HBV^+^/HBV^-^, n=2).**

| **Metabolites** | | **Tumor (T) Tissue (HBV^+^/HBV^-^)** | |  |
| --- | --- | --- | --- | --- |
|  | | **T_HBV+_/T_HBV-_ T_HBV+_/T_HBV-_** | |  |
| **Trans-Vaccenic acid** | 1.78 | | 1.15 | |
| **LPC 18:2** | 1.93 | | 1.13 | |
| **Phosphatidylethanolamine 16** | 0.51 | | 0.93 | |
| **Cholesterol** | 5.18 | | 2.67 | |
| **Palmitoylcarnitine** | 0.45 | | 0.15 | |
| **9-Trans-Palmitelaidic acid** | 3.28 | | 1.19 | |

**Note: T_HBV+_** are the tumor biopsies of two HBV^+^ HCC patients, and **T_HBV-_** are the tumor biopsies of two HBV^-^ HCC patients.

**Tab S5. Metabolic differential between para-tumor tissue of HBV^+^ HCC patients and HBV^-^ counterparts (HBV^+^/HBV^-^, n=2).**

| **Metabolites** | **Para-Tumor (PT) Tissue (HBV^+^/HBV^-^)** | | |
| --- | --- | --- | --- |
|  | **PT_HBV+_/PT_HBV-_ PT_HBV+_/PT_HBV-_** | | |
| **Palmitic acid** | 2.82 | | 1.23 |
| **9Z,12Z-Linoleic acid** | 1.56 | | 1.48 |
| **Trans-Vaccenic acid** | 4.05 | | 1.67 |
| **Lignoceric acid** | 0.51 | | 0.50 |
| **Phosphatidylethanolamine 18** | 0.11 | | 0.59 |
| **α-Linolenic acid** | 0.75 | | 0.89 |
| **Cholesterol** | 11.22 | | 1.42 |
| **Deoxycholic acid** | 1.16 | | 1.21 |
| **Palmitoylcarnitine** | 0.64 | | 0.66 |
| **Oleic acid** | 3.61 | | 1.46 |
| **9-Trans-Palmitelaidic acid** | 2.28 | | 1.39 |
| **Nervonic acid** | 6.87 | | 1.91 |
| **Glycohyodeoxycholic acid** | 0.20 | | 0.08 |
| **Glycocholate** | 0.23 | | 0.16 |
| **Glycodeoxycholic acid** | 0.18 | | 0.11 |
| **Inosine** | | 0.43 | 0.46 |

**Note: PT_HBV+_** are the para-tumor biopsies of two HBV^+^ HCC patients, and **PT_HBV-_** are the para-tumor biopsies of two HBV^-^ HCC patients.
